# FragIdent – Automatic identification and characterisation of cDNA-fragments

**DOI:** 10.1186/1471-2164-10-95

**Published:** 2009-03-02

**Authors:** Dominik Seelow, Heike Goehler, Katrin Hoffmann

**Affiliations:** 1 Institut für Medizinische Genetik, Charité – Universitätsmedizin Berlin, Augustenburger Platz, 13353 Berlin, Germany; 2 Department of Neuropaediatrics, Charité – Universitätsmedizin Berlin, Augustenburger Platz, 13353 Berlin, Germany; 3 Max Delbrück Center für Molekulare Medizin, 13125 Berlin, Germany; 4 Ruhr-Universität Bochum, Medizinisches Proteom-Center, 44801 Bochum, Germany; 5 Max-Planck-Institut für Molekulare Genetik, Ihnestr. 73, 14169 Berlin, Germany

## Abstract

**Background:**

Many genetic studies and functional assays are based on cDNA fragments. After the generation of cDNA fragments from an mRNA sample, their content is at first unknown and must be assigned by sequencing reactions or hybridisation experiments.

Even in characterised libraries, a considerable number of clones are wrongly annotated. Furthermore, mix-ups can happen in the laboratory. It is therefore essential to the relevance of experimental results to confirm or determine the identity of the employed cDNA fragments. However, the manual approach for the characterisation of these fragments using BLAST web interfaces is not suited for larger number of sequences and so far, no user-friendly software is publicly available.

**Results:**

Here we present the development of FragIdent, an application for the automatic identification of open reading frames (ORFs) within cDNA-fragments. The software performs BLAST analyses to identify the genes represented by the sequences and suggests primers to complete the sequencing of the whole insert. Gene-specific information as well as the protein domains encoded by the cDNA fragment are retrieved from Internet-based databases and included in the output. The application features an intuitive graphical interface and is designed for researchers without any bioinformatics skills. It is suited for projects comprising up to several hundred different clones.

**Conclusion:**

We used FragIdent to identify 84 cDNA clones from a yeast two-hybrid experiment. Furthermore, we identified 131 protein domains within our analysed clones. The source code is freely available from our homepage at .

## Background

cDNA clones or the cDNA contained in them are frequently used in yeast two-hybrid assays [1] and hybridisation studies [2]. Although whole clones are employed in some hybridisation studies [3], usually only the insert or a fragment of it is used as a probe. The DNA can be obtained either by amplification of the corresponding plasmid, by insert or vector specific PCR reactions.

While the contents of clones experimentally derived from complex mRNA samples are necessarily unknown, even clones from characterised libraries are in many cases wrongly annotated [4] or might have been mixed up in the laboratory. To draw conclusions from experiments involving such clones, it is inevitable to sequence at least a part of the insert to confirm its identity. A conventional approach is to determine the identities of the clones by sequencing from one or both ends using primers specific to the vector sequence [5-7]. Subsequently, the obtained sequence is matched to annotated sequences in public databases to identify the corresponding mRNA or protein. Although such an initial sequencing is in most cases enough to ascertain the gene encoded by the ORF, it may not cover the coding region completely. In such cases, it is impossible to determine the transcript variant, to detect new transcripts hitherto unknown [8] or to spot 'contaminated' clones that contain sequences not present in the original gene. Especially in yeast two-hybrid screens, it may be crucial to gain knowledge not only of the protein encoded but also of the functional domains covered by the actual clone since interactions are often mediated by protein domains [9].

In these cases, it is therefore indispensable to sequence the whole insert. This can be achieved by successive sequencing reactions with primers aligning at the end of the prior sequence until the vector sequence or a stop codon is reached ("primer walking"). To construct the sequence of the clone, the obtained sequences have to be merged by aligning the overlapping part of the sequences. Alternatively, after the initial sequencing, primers can be designed in advance when the encoded gene is known. If the size of the insert is experimentally determined, the primer design can be restricted to the estimated region.

Whatever method is chosen, the steps performed by the researcher turn out to be a tremendous work when carried out manually for a large number of clones. In addition, the manual alignment of clone sequences to DNA, mRNA or protein databases bears the risk of copy and paste errors as well as the accidental use of different BLAST settings. Also, the generation of suitable insert-specific sequencing primers can require a huge effort when large numbers of clones have to be sequenced, each with multiple primers.

For the systematic analysis of DNA fragments, several bioinformatics tools are freely available. However, some of these tools are either addressed at dedicated bioinformaticians (e.g. EMBOSS [8]) or are specific for other purposes, such as the SABIA system for bacterial genomes (SABIA [9]). Other applications focus on EST sequences, and although they are useful to identify and characterise genes contained in a cDNA clone (AutoFACT[10], EST-PAC[11], OREST[12]), they cannot easily be employed to judge the length of the insert in a cDNA clone nor design new primers needed to sequence the whole insert. EST Express [13], on the other hand, can discriminate between full length and partial sequences and even provides filters for vector sequences but is a comprehensive clone management database which requires a complex installation and might therefore be oversized for projects which comprise less than a few thousand clones and do only require sequence analysis. However, since the use of any such tool is only rarely been mentioned in manuscripts, apparently BLAST analyses are currently mainly carried out either by copying and pasting sequences into one of the BLAST web interfaces or by proprietary software that is not specified in the respective publications and not publicly available. To meet the challenge to analyse more than 80 clones from a yeast two-hybrid experiment, we developed FragIdent, a software that combines the single steps into a single application. Our approach provides a user-friendly interface that guides the researcher through the single steps necessary to identify and to further characterise the cDNA fragments, hence making larger analyses feasible for researchers without any bioinformatics skills.

## Implementation

FragIdent was programmed in Perl/Tk and is freely available. It uses Primer3 [14] for primer design. BLAST analyses are performed via the Internet at the NCBI [15,16]. Basic gene specific data is also obtained from the NCBI. The GeneDistiller database [17] is queried via the Internet to include more detailed information such as protein-protein interactions and reports from the OMIM disease database for the respective genes.

All results are stored in plain text files and can easily be transferred to other applications. Since the graphical output is written in HTML format with PNG images, it can be examined in any web browser without the need of installing our or any other additional software.

## Results and discussion

**Figure 1:**
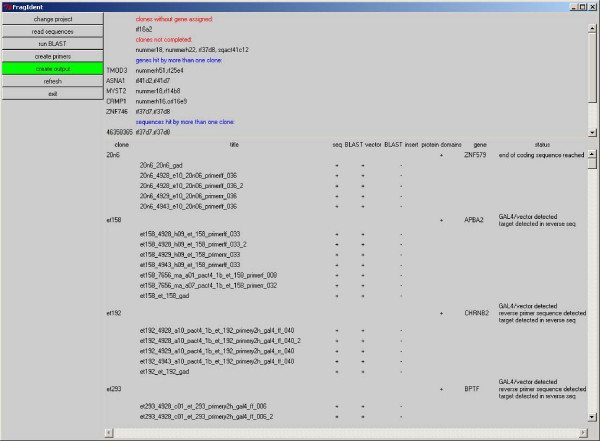
**User interface**. This figure shows the user interface of our software. On the left side, buttons for the possible actions are located. On top of the main frame, possible problems that might require a closer examination (e.g. clones to which no gene could be assigned) are summarised. Below, all available clones with all sequences, BLAST results etc. are listed together with the genes assigned and the current sequencing status.

The program flow resembles the steps in the manual approach. In the first step, FragIdent collects the sequences and blasts them against the target database(s) to identify the ORF represented by the inserts. In the next steps, the alignment of multiple sequences of one clone, the recognition and clipping of the vector sequences, the design of new sequencing primers and the identification of protein domains are carried out. In the final step, all information is integrated in concise output files and is combined with the gene specific information available in public databases. The user is guided through the steps of insert identification and characterisation by a very simple graphical user interface (figure 1).

### Sequence alignment and identification

The software reads sequences in FASTA format and assigns them to the respective clones. The sequences are blasted against the NCBI's databases (with BLASTN) to find the corresponding sequence represented by the insert as well as the vector-specific sequences, thereby assigning the start or end of the insert. Alternatively, a local installation of BLAST can be used.

The search for target ORFs encoded by the cDNA-fragment is performed directly against a (human) mRNA database (or another database as specified by the researcher) with stringent settings (i.e., a high degree of identity is required). For the identification of vector sequence, all available DNA databases are queried with less stringent settings. The default settings of the software (i.e. minimal identity and E values of alignments) and the sequence databases can be easily changed by the users in a configuration file if they desire to query different organisms or focus on genomic clones. Furthermore, it is possible to upload BLAST results obtained by other means or to align single sequences with different parameters. The software can also handle sequences complimentary to the reverse strand and will indicate when the reverse strand was sequenced. BLAST was given preference over BLAT [18], because it allows the explicit use of mRNA databases.

Out of all BLAST hits, the (mRNA) sequence with the best coverage is chosen as 'target sequence'. The software also allows the manual definition of a target gene of a clone. In this case, only BLAST hits against this gene are considered.

FragIdent lists all clones to which no homologous hit can be found in public databases and notifies the researchers of genes covered by more than one clone. If the assigned sequence is not completely covered by the experimentally derived sequences, gaps are indicated graphically and listed.

Our software can use an unlimited number of sequences per clone. Sequences can be added at any time during the analysis. Re-analysis will be restricted to the data that has actually changed.

### Primer design

Additional primers based on the sequence of the cDNA-fragment can be designed automatically using Primer3 with the default settings. Primer design starts around 100 bases before the end of the experimentally confirmed sequence. New primers are created in regular intervals (with a user-specified length) and listed in a file together with their physical positions within the target sequence and the corresponding region (coding sequence or 3' UTR).

### Finding protein domains

After the alignment, the software uses the GI number of the best hit to query Genbank [16] for gene specific information including protein domains. The position of the domains within the protein is then mapped to the coding sequence. The domains are shown in the graphical output and included in the results file. Furthermore, the user is notified whether or not a domain is completely covered by the insert.

### Graphical output and documentation

**Figure 2:**
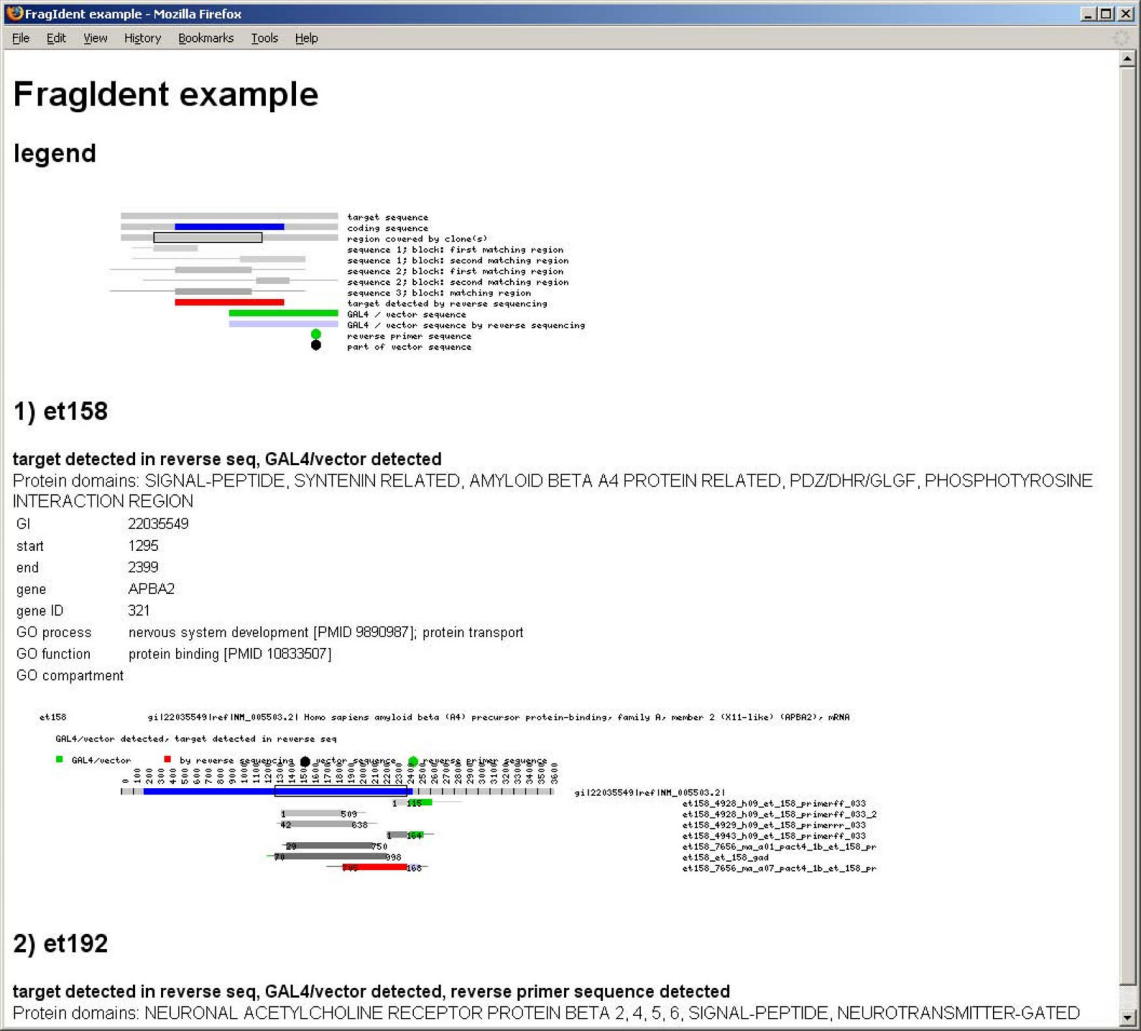
**Alignments – graphical overview**. This screenshot shows the HTML page containing the graphical alignments. The page starts with a short legend followed by the different clones that were studied. This example shows the first clone ('et158'). The output lists the protein domains included in the insert and some gene specific information (more can be found in a separate list). The alignments are displayed in an image below: The target sequence is covered by three forward and one reverse sequences. Three further sequences could not be aligned to the target. The target gene is shown on top of this image as a grey bar with its coding sequence in blue. Below, the different sequences are plotted in different shades of grey with green parts representing the vector sequence. The sequence at the bottom is drawn in red to indicate that its orientation is reversed, i.e. that it was detected by sequencing from the 3' end and therefore determines the 3' end of the insert. The part of the gene that is actually covered by the sequences is emphasised with a box around it. Regions of the target gene not covered by sequences are marked with a thin red line on top of its bar and listed in the text. Protein domains are shown as horizontal purple bars reflecting their position in the cDNA. Below the figure, links to the raw data (sequences and BLAST results) are included.

The alignments and the contained protein domains are displayed graphically in a printable HTML file, together with target gene specific information such as gene symbol and description (figure 2). Links provide fast access to the underlying data (sequences and BLAST results) for each clone. In a further step, information for the (human) genes represented by the inserts is retrieved from public databases and stored as an HTML file. In addition to the identification of the inserts, the software facilitates the documentation of the results, as the information is stored with a clear structure, in plain text files with a standardised format. Within the application only plain text, PNG images and HTML are used and all internal links are relative, hence a project can be shared by simply copying the files onto a web site. It is also possible to resume work on another computer or to join data from different projects by simply copying the files.

## Conclusion

We have presented a data analysis suite to identify cDNA clones that were completely or partly sequenced, to find protein domains covered by the insert and to check the degree of coverage of the insert by experimentally derived sequences. The huge efforts on insert identification and eventually characterisation posed by large-scale studies using cDNA clones make manual approaches cumbersome and error-prone. We have designed our software to overcome the obstacles in an intuitive, user-friendly manner. This automatic approach is aimed at researchers who are not familiar with programming languages and want to analyse their data themselves. The graphical output and the possibility to interact with the analysis at various stages, give the users a high level of control over the identification process. We used this software successfully to analyse 84 cDNA clones from a yeast two-hybrid experiment. All cDNA fragments encoded a human gene. While most of the inserts could be completely sequenced with a single vector-specific primer in the first run, up to 6 further insert-specific primers were needed for longer cDNA fragments. In total, 131 protein domains were mapped onto the cDNA fragments.

## Availability and requirements

Project name: cDNA-Alignment

Project home page: 

Operating systems: all

Programming languages: Perl, Perl/Tk

Other requirements: Primer3, Internet connection

License: free

Any restrictions to use by non-academics: None

## Authors' contributions

DS developed the software, HG provided the clones, and KH coordinated the underlying project and defined the software requirements. DS, HG and KH wrote the manuscript.  KH is supported by Deutsche Forschungsgemeinschaft grant DFG, SFB 577, project A4, and is a recipient of a Rahel Hirsch Fellowship, provided by the Charité Medical Faculty.
